# Untethered Ultra-Wideband-Based Real-Time Locating System for Road-Worker Safety

**DOI:** 10.3390/s24082391

**Published:** 2024-04-09

**Authors:** Aitor Ochoa-de-Eribe-Landaberea, Leticia Zamora-Cadenas, Igone Velez

**Affiliations:** 1 CEIT-Basque Research and Technology Alliance (BRTA), Manuel Lardizabal 15, 20018 Donostia-San Sebastian, Spain; aochoadeeribe@ceit.es (A.O.-d.-E.-L.); lzamora@ceit.es (L.Z.-C.); 2 Tecnun School of Engineering, Universidad de Navarra, Manuel Lardizabal 13, 20018 Donostia-San Sebastian, Spain

**Keywords:** accident prevention, construction sites, real-time tracking, RTLS, safety, UWB

## Abstract

In order to reduce the accident risk in road construction and maintenance, this paper proposes a novel solution for road-worker safety based on an untethered real-time locating system (RTLS). This system tracks the location of workers in real time using ultra-wideband (UWB) technology and indicates if they are in a predefined danger zone or not, where the predefined safe zone is delimited by safety cones. Unlike previous works that focus on road-worker safety by detecting vehicles that enter into the working zone, our proposal solves the problem of distracted workers leaving the safe zone. This paper presents a simple-to-deploy safety system. Our UWB anchors do not need any cables for powering, synchronisation, or data transfer. The anchors are placed inside safety cones, which are already available in construction sites. Finally, there is no need to manually measure the positions of anchors and introduce them to the system thanks to a novel self-positioning approach. Our proposal, apart from automatically estimating the anchors’ positions, also defines the limits of safe and danger zones. These features notably reduce the deployment time of the proposed safety system. Moreover, measurements show that all the proposed simplifications are obtained with an accuracy of 97%.

## 1. Introduction

Occupational accidents are of major concern for workers, their families, and companies. However, according to Eurostat, over 2.8 million non-fatal accidents and 3347 fatal accidents were reported in 2021 in the European Union of 27 states (EU-27) [1]. Among all activities, the construction sector had the highest incidence rate of non-fatal accidents: 3151.9 accidents per 100,000 persons employed [2]. Thus, the construction sector has an urgent need to improve its safety proceedings.

Among all types of construction sites that exist, road construction is a particular case in which workers are exposed to serious risk of death due to heavy vehicles driving fast nearby. In order to guarantee a safe working environment, so far, the safety awareness of workers and drivers has been increased by means of personal protective equipment (PPE), informative sessions, training, and traffic control devices [3]. However, human behaviour is difficult to control [4] and the safety procedures are not always correctly followed. This risk can be avoided with the help of behaviour-based safety (BBS), which is designed to intervene and modify unsafe human behaviour. BBS is an approach of systematic application of psychological research into human behaviour. The works of [5,6] showed that, for the successful application of BBS in construction sites, constant surveillance of workers is needed. Normally, this surveillance is performed by other humans, so the collection of data is highly inefficient and can also be inaccurate due to the subjective judgments needed [7]. Thus, BBS cannot be the only approach in construction sites to improve the safety of workers.

As an alternative, the application of newly emerging technologies in personalised safety monitoring could be used, a solution that has not been completely explored [8]. There are some works that have studied the feasibility of warning workers if a distracted driver enters the working zone [9,10]. Nevertheless, to our knowledge, no previous work has proposed any solution for distracted workers leaving a safe zone in a road construction environment. A good example in the case of road and railway maintenance could be the use of a real-time locating system (RTLS) that detects if workers remain in the safe working environment. However, these types of solutions may have some drawbacks because of the difficulty of deploying an RTLS [11] and the dynamic nature of construction environments [12]. Most safety systems based on RTLS [13,14,15,16,17,18,19,20,21,22,23,24,25,26,27,28,29] need to deploy some fixed sensors with known positions around the working environment and define safe and dangerous zones. Some time has to be spent fixing sensors where needed, measuring their positions and introducing the limits of the safe and danger zones to the system. All these tasks add preparation time to the working project. Moreover, as construction environments are highly dynamic, adjustments must be made during the lifetime of projects and the time spent preparing an RTLS is multiplied by the number of changes made. Another drawback of adding an RTLS to construction sites is that they suppose extra elements that can add difficulty to the tasks to be performed. If, for example, cables are added to power sensors, workers may trip and fall. Any safety system for construction sites should be fast and simple to use and interfere as little as possible with the work.

Apart from the general requirements of construction environments, the use case of an RTLS in road and railway maintenance also demands high accuracy. If a worker unintentionally leaves the delimited safe zone but the RTLS considers them to be in the safe zone, the worker will not receive any alarm. Thus, the worker will be at serious risk of suffering an accident. The other way around can also have negative consequences, since a worker receiving unnecessary alarms will consider the safety system unreliable and will stop paying attention to it.

This paper proposes a novel safety system for construction sites especially useful in road and railway maintenance. Our safety system is an RTLS that uses ultra-wideband (UWB) technology for the safety assessment of workers. Unlike previous works [9,10], our proposal solves the problem of accidents caused by distracted workers that leave the safe zone instead of focusing on distracted drivers that enter this safe zone. Thanks to the high accuracy of UWB technology, it can reliably tell if a worker is inside or outside a predefined safe zone. If the worker is not in the safe zone, a warning or an alarm is given, depending on the seriousness of the situation. Because of the special characteristics of construction environments, the fixed UWB sensors are placed inside safety cones and the moving sensors in the workers’ hard hats. As a result, safety cones act as fixed sensors of the RTLS and safety limits at the same time, which simplifies the deployment of the system. Our proposed safety system also avoids adding congestion to construction sites, as safety cones are common in them. Moreover, because of the low power demand of UWB sensors in safety cones, they can be powered by photovoltaic cells, avoiding extra congestion by cables. Additionally, for further simplification of the deployment of the safety system, a self-positioning approach of UWB fixed sensors is proposed. This self-positioning approach, apart from estimating the positions of the anchors’ coordinates, also automatically sets the limits of the safe, warning, and danger zones without needing an extra communication link among anchors.

The sections of the article are organised as follows. Section 2 reviews all the work involving RTLS for safety applications in construction environments. Section 3 describes the proposed system in depth. Section 4 describes the performed experiments for the evaluation of the safety system. Finally, Section 5 discusses the obtained results and Section 6 gives the final conclusions of the work.

## 2. State of the Art

Global navigation satellite system (GNSS) is one of the most well-known positioning systems. Nevertheless, this technology has significant limitations in indoor and urban environments [30], where many construction sites are located. Thus, since 2012, research on RTLS technologies has gained relevance for safety management in construction environments [31]. The most common technologies for this purpose are computer vision, radio frequency identification (RFID), bluetooth low energy (BLE), and UWB.

In recent years, there has been an increase in research works using computer vision in construction environments due to their abundance of visual data and the recent advancements in this area [32]. In the works of [33,34,35], equipment and workers were successfully tracked using computer vision. However, all these works had a common problem to deal with: occlusions. When an entity is tracked with cameras, it may temporarily hide behind an object and be lost, which leads to failure. In order to mitigate this drawback, the research works of [33,34,35] suggest carefully choosing the positions of cameras, so that their field of view covers as much as possible of the working place. Nevertheless, selecting adequate positions for the cameras demands some planning time and extra infrastructure to place them where desired. Moreover, the construction environment is highly dynamic, so the chosen camera positions might not be adequate during all the phases of the project.

Refs. [36,37] proposed using a combination of tracking and detection algorithms to deal with occlusions. When the track of a worker is lost, it can be detected again. However, there is still high uncertainty during the time of an occlusion in systems based on computer vision, as the position of the tracked object is completely unknown.

Radio frequency-based RTLSs are less affected by occlusions and the identification of each sensor is an easy task [16]. The latter feature is crucial if an individual alarm must be sent to the person who is detected to be in danger.

An example of radio frequency-based technologies is RFID. This technology has been intensively researched for construction environments in the last decade due to its cheap and flexible approach to identify individual people and devices [38]. RFID consists of tags and readers. Tags emit a signal which is later read by the readers to calculate the distances between sensors [39]. The main setback of RFID technology is its accuracy. The works of [40,41] proposed using RFID in construction environments, but only to send proximity alerts between workers and equipment [40] or to calculate the rough position of workers [41]. However, if the actual positions of workers need to be determined with centimetre-level accuracy, RFID is not the most suitable technology [13,42]. In the case of [42], some danger zones were defined in a construction environment with a false alarm rate of 87% if a worker stood 1 m away from one of these zones. With such a high probability of receiving false alarms, workers might end up not paying attention to the safety system and ignoring alarms.

BLE is another radio frequency-based technology that has recently gained relevance as a safety system [14,15,16,18,43,44]. BLE shows advantages such as its low cost and power consumption. It also has a high availability, as most smartphones use this technology. Examples of BLE in construction sites can be found in [14,15], where several sensors were placed in the construction sites of a building and a petrochemical plant, respectively. Both construction sites were full of BLE sensors; in the case of [14] 39 sensors were placed in a 39 × 27.5 m construction site, and in the case of [15], 34 sensors in a 2736 $m^{2}$ site. The positions of all these sensors must be measured and introduced to a software manually. Such complex systems could add significant preparation time to the construction project. Moreover, the most important drawback of BLE-based systems is their poor accuracy. Generally, BLE can have positioning errors between 1 m and 10 m [15].

The newest version of BLE, version 5.1 and 5.2, called BLE-AoA, offers enhanced accuracy. The ability to estimate the angle of arrival (AoA) improves the accuracy of these systems. In fact, the work of [16] proposed an approach where BLE-AoA is used. However, only 60% of measured estimates had submetre accuracy.

Another radio frequency-based technology for RTLSs is chirp spread spectrum (CSS). The authors of [17] proposed an RTLS for safety purposes in a construction site using CSS. The obtained positioning accuracy was 0.868 m, which is quite poor if the RTLS must determine if a worker is in a safe or danger zone.

As explained before, most systems using RFID, BLE, or CSS technologies do not always offer the necessary positioning accuracy for safety applications. As an alternative, these technologies can be combined with other sensors for enhanced accuracy. For example, the authors of [18] proposed a fusion between inertial sensors and BLE. Their system consisted of four fixed BLE sensors on tripods and tags with inertial sensors on the hard hats of workers, obtaining a mean positioning error of 0.32 m in a bridge construction site. In spite of the acceptable positioning performance, their proposal still requires a complex deployment process.

For applications of high accuracy, UWB technology is the best radio frequency-based alternative in construction environments [45]. Compared to other radio frequency-based technologies, UWB presents higher immunity to multipath effects [46], which is advantageous in environments of high congestion such as construction sites. Thanks to the high accuracy of UWB, the deployment of an RTLS can be simplified.

In the works of [19,20,21], different methods for the evaluation of UWB systems were defined for construction environments. The most typical unfavourable conditions of construction environments were simulated, such as the presence of construction equipment (metallic objects) in the proximity of sensors. The authors of [22] ran numerical simulations to test the applicability of UWB technology for safety systems. Some other research papers went a step further with the implementation of UWB-based RTLSs in real construction environments [23,24,25,26]. The accuracies of the RTLSs were suitable for the proposed applications, but the tested systems presented some downsides that need to be corrected. A typical UWB system, such as those of [19,20,21,22,23,24,25,26], consists of a set of UWB anchors and tags. The anchors are fixed sensors at known locations that perform time difference of arrival (TDoA) measurements with the tag. For these measurements, the anchors need an accurate clock synchronisation obtained with a wired communication. The TDoA information is then sent to a processing hub via ethernet cables to calculate the position of the tags. The first disadvantage of these systems is the use of cables, since if cables are placed incorrectly, a worker may trip and fall. There is also the possibility of accidentally damaging the cable, with the consequence of a poorer performance due to the lack of a sensor. Thus, some time has to be spent to decide the correct placement of cables. In addition to that, as the construction environments are highly dynamic, the need to change the initial placement of anchors can be very common. A tethered configuration slows these adjustments.

As a solution, different works present untethered UWB systems for their use in construction sites [27,28] or as a safety system with a geofencing strategy [29]. However, the removal of synchronisation cables can come at the expense of poorer accuracy [28].

If good accuracy is needed without the use of a wired communication, there are other UWB-based RTLSs that use time of arrival (ToA) measurements without the need for a time synchronisation [47,48,49]. Nevertheless, RTLSs using ToA still present some deployment complexities that have to be overcome. The suitable positions for anchors must be selected and their positions must be measured. Moreover, if used as a safety system, the coordinates of danger zones must also be measured and introduced to the system [49].

For faster deployment, there are several works that have proposed the automatic calculation of the positions of anchors [50,51,52,53]. These works need the anchors to communicate with each other, which increases their power consumption. This increase in power demand can be acceptable if anchors are plugged in to the electrical network. In construction sites, however, it is important to avoid wired connections, so anchors should be battery-powered. Thus, reduced power consumption becomes crucial. Another setback of [50,51,52,53] is that a communication link must be added to a central computer to decide when the moment of calibration is, and when the moment of normal operation begins. The need for a central computer can be burdensome, especially in a large road construction site of hundreds of metres.

A more flexible self-positioning approach is that proposed by [54], which uses a simultaneous localisation and mapping (SLAM) algorithm by fusing data of a UWB tag with an inertial measurement unit (IMU). Nevertheless, this algorithm adds computational complexity and increases the power consumption because of the IMU measurements. Moreover, if a self-positioning approach is to be used with anchors that delimit a safe zone, a polygon must be created with the positions of anchors. In this case, the order in which these positions are joined is crucial to generate the polygon. The automation of this process still remains a research gap with UWB technology.

## 3. Description of the Proposed Safety System

In this work, the proposed UWB-based safety system is intended to be used in dangerous construction environments where a safe zone must be defined for work. This is a typical case in the maintenance of roads and railways, where vehicles can be driving at high speeds close to the working place. The developed safety system can locate the workers of those environments and give them an alarm if necessary.

**Figure 1:**
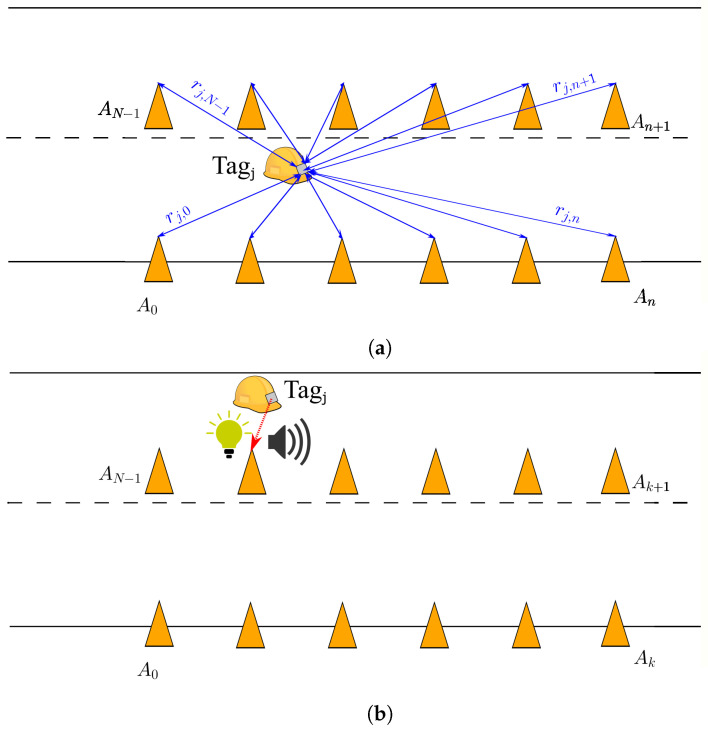
Architecture of the proposed safety system. (**a**) Distance calculation between cones and tags, (**b**) operation under unsafe conditions.

**Figure 2:**
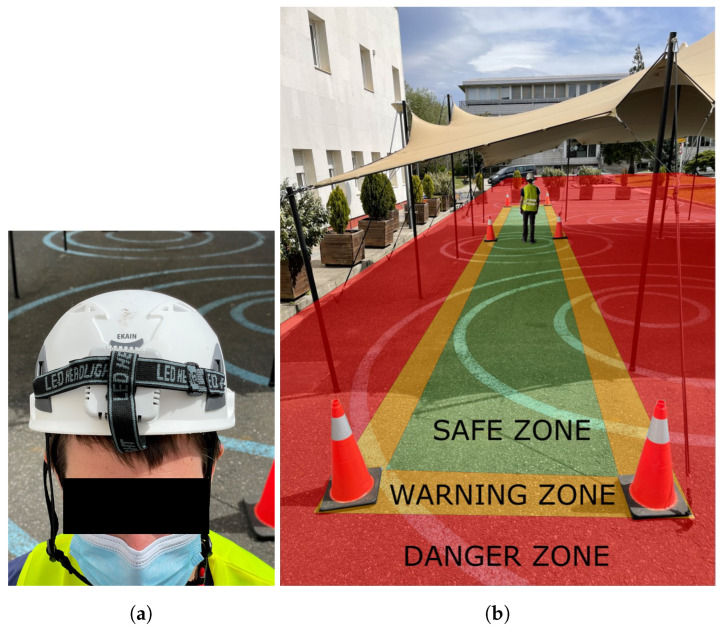
(**a**) Tag on hard hat of worker and (**b**) deployment of safety system with marked zones.

The architecture of the described safety system is presented in Figure 1. Let us consider a use case where maintenance work is performed on a road. In this case, workers occupy one of the lanes of the road and it becomes unavailable for normal traffic. However, vehicles are still allowed to use the other lane, so a safe working environment must be defined for workers. Usually, the safe working environment is delimited with safety cones. In this work, a safety system is proposed that adds a UWB-based RTLS to improve the safety awareness of workers. The traditional safety cones are replaced by intelligent cones, which contain UWB anchors. These anchors do not need any cables for powering them, as their low power demand allows them to be powered by photovoltaic panels. The workers wear a hard hat containing a UWB tag, as shown in Figure 2a.

During the operation, each pair of UWB anchor and tag communicate with each other so that the distance between them is estimated, as depicted in blue in Figure 1a. The tags collect all these data to calculate the positions of workers in real time. This information is then used to estimate if workers are in danger or not. If a worker wearing a tag is detected to be unsafe, their tag orders the closest cone to send a warning signal or an alarm, as depicted in red in Figure 1b. The type of signal depends on the severity of the situation. In the case of a warning, the closest cone to the unsafe worker turns on a light. A visual stimulus is easy to detect and non-intrusive at the same time. Thus, if a warning is given, the worker knows that they may be in danger, and at the same time, they can decide to remain where they are if they consider themselves to be safe. In the case of an alarm, the closest cone to the unsafe worker sends an acoustic signal. Depending on the situation, these signals could be difficult to see or hear. That is why the proposed system has been designed in such a way that the light is bright enough to be used on sunny days. If, in a certain situation, this is not enough, a blinking light can be used, as signals in motion are easier to detect [55]. If the work area were too noisy, a wristband could be added to the workers that sends a vibro-tactile alarm instead of an acoustic signal, as by [16,56].

**Figure 3:**
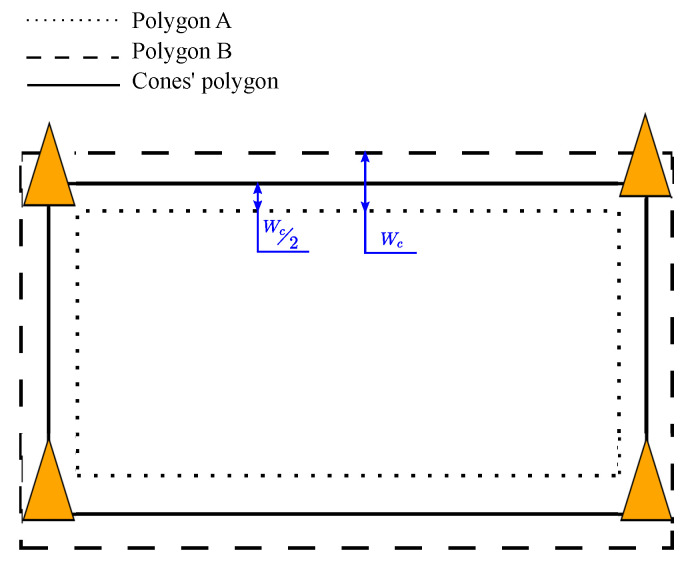
Definition of safe, warning, and danger zones with the cones’ polygon.

The safety conditions of workers are determined by defining three different zones on the plane: the safe zone, warning zone, and danger zone. Those zones have been drawn on the photograph of Figure 2b. The area inside the polygon of cones drawn in green is the safe zone. The outer part of the polygon of cones, or the danger zone, is drawn in red. There is also a warning zone drawn in orange as wide as the base of a cone, which is close to the average shoulder width of an adult human. For the correct definition of all zones, first the positions of cones must be known by the safety system and an identifier number must be given to each cone. As illustrated in Figure 3, the positions of cones define the polygons that represent all zones. Given a cone width of $W_{c}$, two polygons are defined: polygon A and polygon B. Polygon A is the result of reducing the limits of the cones’ polygon by $W_{c}/2$, while polygon B is obtained by increasing the limits of the cones’ polygon by $W_{c}/2$. The area contained by polygon A is called the safe zone and the area outside polygon B is the danger zone. The area of polygon B not contained by A is the warning zone. With the definition of the warning zone, workers can be warned when they are at risk of leaving the safe zone without the necessity of giving an alarm.

Even though only four cones are shown in Figure 3, the safety system supports a larger number of cones. Moreover, not all cones need to be within communication range of each other. It is only necessary that a tag be in communication range of at least four cones. It is important to note that in a road construction site, cones should be separated by about 6 m [57], a distance that is well below the communication range of a UWB system. In fact, it is even possible to combine the safety cones of our system with regular safety cones to obtain a more cost-efficient deployment.

After the deployment of the proposed safety system, it is ready to be used. The characteristics of the UWB sensors used by our safety system are shown in Table 1.

The UWB anchors and tags are from SafeLoc Systems S.L. [58] and follow the IEEE 802.15.4a standard [59]. These devices are calibrated during production by the manufacturer, so there is no need for calibration during the deployment of the safety system. The tags on the hard hats of workers communicate periodically with the anchors in the safety cones. This communication allows the estimation of the positions of workers and informs them in real time if they are working in a safe working environment or not. Depending on the situation, a warning or an alarm can be given. If a tag is in the safe zone, nothing is done, since the worker wearing that tag is working safely. If the tag is detected to be in the warning zone, a warning is given. If the tag is detected to be in the danger zone, an alarm is triggered. In the proposed system, the tag is responsible for indicating to the closest cone that a warning or danger signal should be sent. Thus, the proposed safety system does not implement multi-hop communication. However, this feature could be added if alarms or warnings are to be sent to a potential central server.

**Figure 4:**
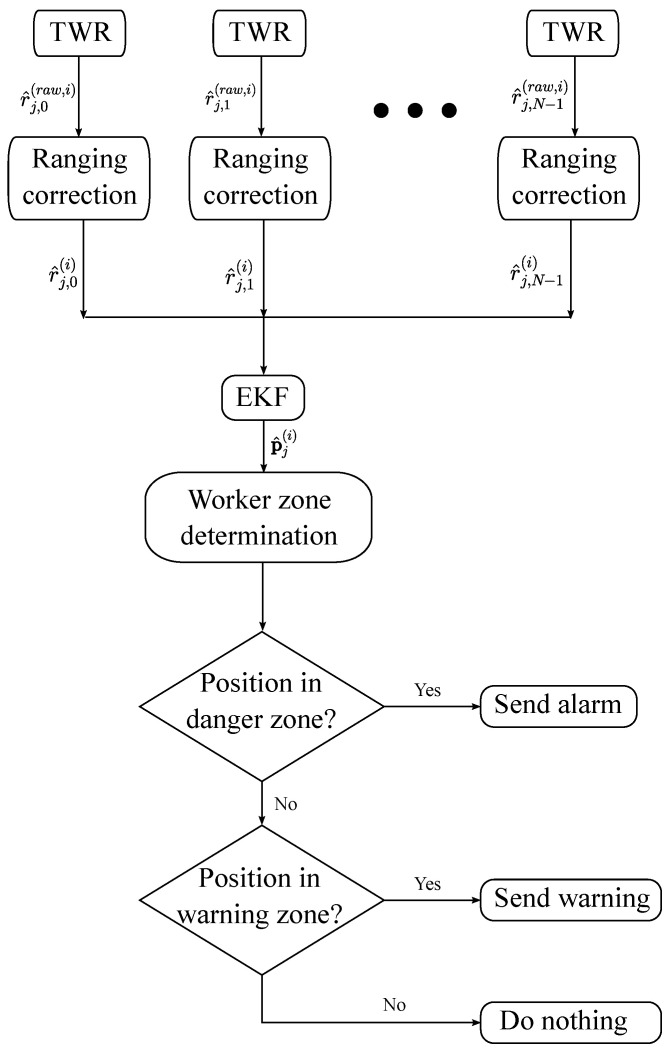
Flow chart of the operation of the safety system.

In Figure 4, a flow chart of the operation of our safety system is shown.

This flow chart considers the process for tag *j* at time step *i* and the subscript *n* is used to refer to any cone ranging from 0 to N−1. First, the ranging estimates between each cone and tag ${\hat{r}}_{j,n}^{\left(raw,i\right)}$ are measured by means of the two-way ranging (TWR) algorithm [59]. A time-division multiple access (TDMA) scheme is used to avoid collisions. However, ranging estimates may contain errors that have to be corrected. Thus, the same method as by [60] is used to correct the ranging estimates. In the flow chart, the corrected ranging estimates are represented as ${\hat{r}}_{j,n}^{\left(i\right)}$. The corrected ranging estimates are then used in an extended Kalman filter (EKF) algorithm as in [60] to calculate the positions of the tags ${\hat{p}}_{j}^{\left(i\right)}$. In order to decide whether or not to warn a worker, it has to be determined in which zone they are. Previously, in the deployment of the safety system, two polygons have been defined based on the cones’ polygon. To determine if a tag’s position is inside a polygon or not, a similar algorithm to that proposed by [61] is used. If the position ${\hat{p}}_{j}^{\left(i\right)}$ is detected to be in the danger zone, an alarm is sent, if ${\hat{p}}_{j}^{\left(i\right)}$ is in the warning zone, a warning is sent, and if ${\hat{p}}_{j}^{\left(i\right)}$ is in the safe zone, nothing is done.

With such a configuration as in Table 1, a position estimate can have a maximum delay of 200 ms. This means that if a worker walks straight out of the safe zone, the safety system will detect that worker to be out of the safe zone 200 ms after crossing the limit. If we assume that the worker walks at 5 km/h, they will be detected 27.8 cm after leaving the safe zone. Thus, if a warning zone of about the average shoulder width of adult humans is used, the selected ranging rate of 5 Hz guarantees that the worker will receive a warning before reaching the danger zone. With the selected update rate, up to ten tags can be monitored, which is more than enough for the number of workers usually found on many construction sites.

The resulting safety system of this work presents several advantages compared to other traditional systems. Compared to a typical safety system, where only some safety cones are deployed around the working environment, the addition of UWB sensors improves the safety awareness of workers since they are warned if they leave the safe zone. Moreover, compared to other RTLS-based safety systems, our safety system is easy to deploy, more flexible, and less intrusive. First, the use of the TWR algorithm avoids the need for any clock synchronisation among UWB anchors inside safety cones. Thus, our proposed safety system does not need any cables among cones that could interfere with the path of a worker or a piece of equipment. Second, the used UWB anchors consume around 200 mA, so photovoltaic cells can be used to power them instead of adding cables. Moreover, selecting appropriately the batteries of anchors, the system can also work during the night. The added cost of using the proposed safety system with photovoltaic cells and batteries is really affordable, especially if we compare it with the more than EUR 1 million that an accident can cost an employer [62]. Third, having the anchors of the safety system in safety cones reduces the time of deployment, since only those cones must be placed where desired, without thinking where to put the UWB anchors. The reduction in time of deployment is also crucial because of the dynamic nature of construction sites. During the lifetime of a construction project, many changes must be made, so the deployment of the safety system will be carried out several times. Thus, any reduction in preparation time is multiplied by the changes made during the project. Finally, the highly congested nature of construction environments makes it difficult to add extra material for an RTLS. However, safety cones are usually an available element on construction sites, so our proposed safety system does not occupy extra space.

### Deployment of Safety System

The proposed safety system has an easy and simple deployment, as only safety cones must be placed where necessary and then their positions must be measured. The simplest way of measuring their positions is with a robotic total station. However, when there is a lack of such equipment, our proposed safety system adds the possibility of self-positioning cones. Thus, the deployment of the safety system remains simple even if no accurate equipment is available to measure the positions of cones.

**Figure 5:**
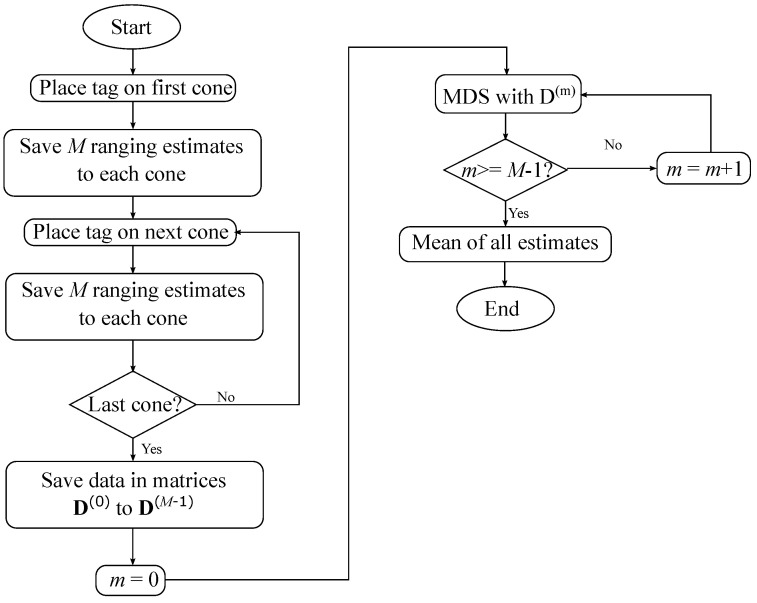
Flow chart of self-positioning method.

A flow chart of the self-positioning process is shown in Figure 5.

In this process, a special tag called the calibration tag is used calculate the distances between each pair of cones. The calibration tag is represented with the subscript j=0. First, the calibration tag is placed for a period of time *T* on each cone. The period of time *T* allows the system to obtain *M* ranging estimates between the calibration tag on the position of a cone and the rest of cones. The flow chart of Figure 5 considers *N* cones going from 0 to N−1. The *m*th ranging estimate between cone *n* and the calibration tag placed on the position of cone *l* is represented as $r_{0,n}^{\left(m,p_{l}\right)}$. The variable *m* takes any value between 0 and M−1 and the variables *n* and *l* take any value between 0 and N−1, given that n≠l.

Once the calibration tag has been placed on each cone, the *m*th distance estimate between cones *n* and *l*, $d_{n,l}^{\left(m\right)}$, is calculated as a mean of the ranging estimates from cone *n* to the calibration tag on cone *l* and from cone *l* to the calibration tag on cone *n*:

(1) $$d_{n,l}^{\left(m\right)}=\frac{{\hat{r}}_{0,n}^{\left(m,p_{l}\right)}+{\hat{r}}_{0,l}^{\left(m,p_{n}\right)}}{2}.$$

With this process, there are *M* distance estimates between each couple of cones, which are necessary to calculate their positions. The distance estimates are saved in *M* different dissimilarity matrices:

(2) $$D^{\left(m\right)}=\left[\begin{array}{ccccc} 0 & d_{0,1}^{\left(m\right)} & d_{0,2}^{\left(m\right)} & \cdots & d_{0,N-1}^{\left(m\right)}\\ d_{1,0}^{\left(m\right)} & 0 & d_{1,2}^{\left(m\right)} & \cdots & d_{1,N-1}^{\left(m\right)}\\ d_{2,0}^{\left(m\right)} & d_{2,1}^{\left(m\right)} & 0 & \cdots & d_{2,N-1}^{\left(m\right)}\\ \vdots & \vdots & \vdots & \; & \vdots \\ d_{N-1,0}^{\left(m\right)} & d_{N-1,1}^{\left(m\right)} & d_{N-1,2}^{\left(m\right)} & \cdots & 0 \end{array}\right].$$

Each dissimilarity matrix is used to calculate the positions of all cones by means of the multidimensional scaling (MDS) algorithm [63].

**Figure 6:**
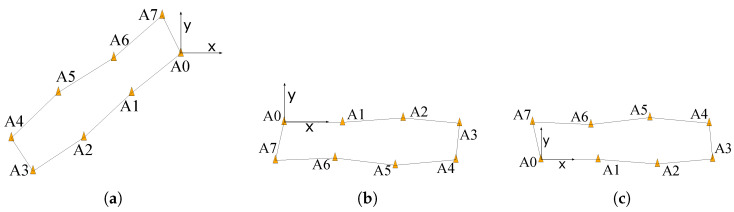
Application of constraints to cone positions with an example of eight cones. (**a**) Constraint 1. (**b**) Constraint 2. (**c**) Constraint 3.

Finally, the positions of the cones are estimated as the mean of all results obtained with those *M* different dissimilarity matrices. However, as the MDS algorithm only determines the relative positions of the cones, the obtained results have six degrees of freedom (DoF) in space, so the different groups of cone position estimates may have different origins of coordinates. Taking into account that our proposed safety system, unlike traditional positioning systems, favours a linear growth of the infrastructure, three geometric constraints are applied:

- Constraint 1: The first cone where the calibration tag has been placed is assumed to be the origin of coordinates. Figure 6a illustrates this constraint, where cone A0 corresponds to the first cone.
- Constraint 2: The second cone where the calibration tag has been placed is assumed to only be displaced in the positive direction of the horizontal axis, i.e., x>0, y=0, and z=0. Figure 6b illustrates this constraint, where cone A1 corresponds to the second cone.
- Constraint 3: The last cone where the calibration tag has been placed is assumed to have a positive *y* coordinate and z=0. Figure 6c illustrates this constraint. As the example considers four cones, cone A7 corresponds to the last cone.

Note that as shown in Figure 6, there is no need for the safety cones to form a rectangle. The proposed self-positioning method can handle any kind of polygon shape with any number of cones.

The calculated cones’ positions and identifiers are stored in the calibration tag. This tag is also responsible for sending the cones’ position data and identifiers to the tags on the hard hats of workers so that the operation can begin. The cones’ positions and identifiers are the only information needed by the system to start working.

This proposed self-positioning process presents many advantages, especially interesting for a safety system on road construction sites. First, there is no need for communication among anchors, which reduces the power consumption. Thus, the safety system can operate with photovoltaic cells or batteries instead of plugging anchors in to a central power supply. Second, anchors do not need to be connected to a central computer that orders the start of the calibration process or the normal operation. In our case, anchors simply communicate with tags even in the calibration phase. Finally, by placing the calibration tag on each anchor, the worker easily decides in which order the positions of anchors shall be joined to generate the cones’ polygon. This approach is less error-prone, as the worker does not have to manually introduce the identification number of each anchor in a central software.

## 4. Experiments

The performance of the proposed safety system was assessed with several experiments. In this section, the performed experiments as well as the used evaluation methods are described.

### 4.1. Indoor Experimental Set-Up

**Figure 7:**
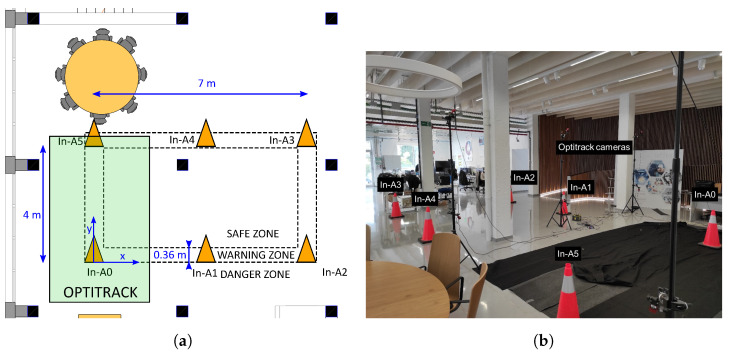
(**a**) Drawing of the deployed set-up. (**b**) Photograph of the experimental environment.

The proposed safety system was tested in a laboratory at the authors’ institution, trying to emulate the typical case of a maintenance operation on a straight road or railway. These construction environments have a similar shape. They cover a small width of about 4 m per lane [64] or track [65] and expand along the road or railway, forming a rectangle. As shown in Figure 7a, six cones were placed, defining a rectangle of 7 × 4 m.

Around the limits of that rectangle, a warning zone of 0.36 m was defined, which coincides with the width of the base of the used cones. The origin of coordinates was set on the cone In-A0 and Table 2 shows the real horizontal coordinates of all the cones.

Part of this zone was covered by an Optitrack motion capture system, which is able to track the movements of the person with millimetre-level accuracy. Because of the high accuracy of the motion capture system, it was used as ground truth to evaluate the performance of our proposed safety system.

In Figure 7b, a photograph of the experimental area is shown. The used cones with the UWB anchors inside can be seen in the photograph. There were some obstacles, such as columns or tripods to hold the Optitrack cameras, which could affect the measurements of UWB sensors. However, on a construction site, it is not expected to have a clear line of sight between sensors, so this area can give us an idea of how our proposed system could work in a real environment.

Although the real positions of the cones were measured before testing the proposed safety system, the self-positioning mode of the proposal was also used to compare how well it performs with real or estimated cone positions. The self-positioning of the safety system is performed by placing a tag on each safety cone, as explained in Section 3.

In order to test the proposed safety system, several measurements were taken. First, as shown in Figure 2a, a 1.78 m tall person wearing a hard hat with the tag walked different paths in the zones defined by the safety system. Note that informed consent was obtained from the participant for the publication of their images and the results of the experiments. The objective of these measurements was to test the positioning accuracy performance of the safety system with a moving tag. The tag on the hard hat was always oriented in the forward walking direction, while all anchors in the cones were oriented towards the inside of the polygon delimited by the anchors (cones). Thus, the relative anchor–tag angles were different during the experiments, and line-of-sight (LOS) and non-line-of-sight (NLOS) conditions occurred. The second type of measurements consisted of a person wearing a hard hat and standing at three different points close to the limits of the cones’ rectangle. This was carried out to evaluate the classification performance of the safety system while guaranteeing a similar number of samples in the safe, warning, and danger zones. In Table 3, the chosen points are described. In these static experiments, the tag always faced the negative direction of the y-axis drawn in Figure 7a. Similarly to the dynamic experiments, the relative anchor–tag angles were different in each measured point. Thus, the experiment considered variability in the anchor–tag orientation.

In the experiments, the tracking of the tag was performed with the real cone positions and all the ranging and positioning estimates were saved for further analysis. The saved ranging estimates were then used in simulations along with the calculated cones’ positions by the self-positioning approach. The simulations gave, as a result, the performance of the proposed safety system with the self-positioning approach. This way, the same data were used to compare the performance of the safety system with real and estimated cone positions.

### 4.2. Outdoor Experimental Set-Up

**Figure 8:**
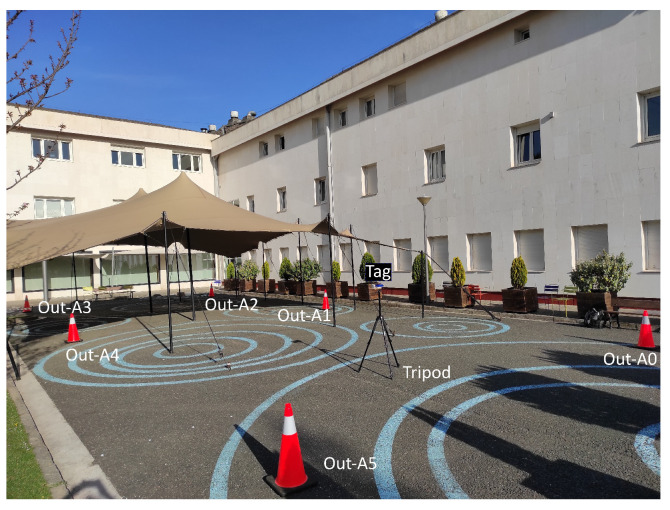
Set-up in the outdoor environment.

The proposed safety system was also tested in another environment to test the repeatability of the results with a different deployment. The chosen place was outside the building of the authors’ institution, where a road has been adapted for pedestrian use. This way, the tests were performed on a road while guaranteeing the safety of the people taking the measurements. A picture of the set-up can be seen in Figure 8.

Similar to the indoor scenario, the real positions of cones were measured but the self-positioning approach was also used for a comparison between the proposed system with self-positioned cones and manually measured cones. The real positions of cones are shown in Table 4.

As it can be observed, the width of the rectangle was 8 m, which could be the case in maintenance of a two-lane road. There is also a distance of 10 m between two consecutive cones in the x direction. In a real application, more regular cones could be placed in between to add a visual reference of the safety limits.

The positioning accuracy of the safety system was evaluated by placing the tag on a tripod with a height of 1.45 m at 12 different points inside and outside the zone delimited by the cones. The chosen points are presented in Table 5.

The last three points, Out-P10, Out-P11, and Out-P12, were also used to test the classification performance, since these are the three closest points to the limits between zones. The tag always faced the positive y direction of the local coordinate system during the measurement.

### 4.3. Evaluation Methods

The positioning accuracy of the proposed safety system was measured by comparing the estimated positions with the real ones. In the indoor environment, the Optitrack motion capture system gave the true path, and in the outdoor environment, the predefined known points where the tag *j* was placed. For each estimated position ${\hat{p}}_{j}^{\left(i\right)}={\left[\begin{array}{cc} {\hat{x}}_{j}^{\left(i\right)} & {\hat{y}}_{j}^{\left(i\right)} \end{array}\right]}^{T}$, its error was calculated as

(3) $$\epsilon_{j}^{\left(i\right)}=\sqrt{{\left({\hat{x}}_{j}^{\left(i\right)}-x_{j}^{\left(i\right)}\right)}^{2}+{\left({\hat{y}}_{j}^{\left(i\right)}-y_{j}^{\left(i\right)}\right)}^{2}},$$

with $\epsilon_{j}^{\left(i\right)}$ being the positioning error at time step *i*, ${\hat{x}}_{j}^{\left(i\right)}$ and ${\hat{y}}_{j}^{\left(i\right)}$ the two-dimensional (2D) coordinates of the estimated position, and $x_{j}^{\left(i\right)}$ and $y_{j}^{\left(i\right)}$ the 2D coordinates of the real position. Once all the positioning errors were calculated, the system was evaluated with the mean positioning error μ, standard deviation of the positioning error σ, positioning root mean square error (RMSE) [66], and the maximum positioning error $\epsilon_{max}$.

The classification performance was evaluated by means of a confusion matrix [67], which is a useful tool to analyse the performance of a classifier. In the case of our proposed safety system, we evaluated how well a worker was detected to be in the safe, warning, or danger zone. As our proposed safety system considers these three different cases, a 3 × 3 confusion matrix was used for its evaluation. Thus, the evaluation of the classifier was performed in three steps, considering, in each case, a positive in every step. For example, in the first step, the samples in the danger zone were considered positives, while the samples in the warning and safe zones were negatives. In the second step, the samples in the warning zone were considered positives and the samples in the danger and safe zones negatives. In the third and last step, the samples in the safe zone were considered positives and the samples in the danger and warning zones negatives. After these three steps, the total number of true positives, false positives, true negatives and false negatives were summed up to evaluate the overall performance. The accuracy and $F_{1}$ score [67] were used to evaluate the performance of the classifier.

## 5. Results

### 5.1. Positioning Accuracy in Indoor Environment Using Real Cone Positions

The positioning accuracy of the proposed safety system was first assessed in an indoor environment. In this environment, an Optitrack system was available and was used to obtain the true path in the dynamic experiments.

**Figure 9:**
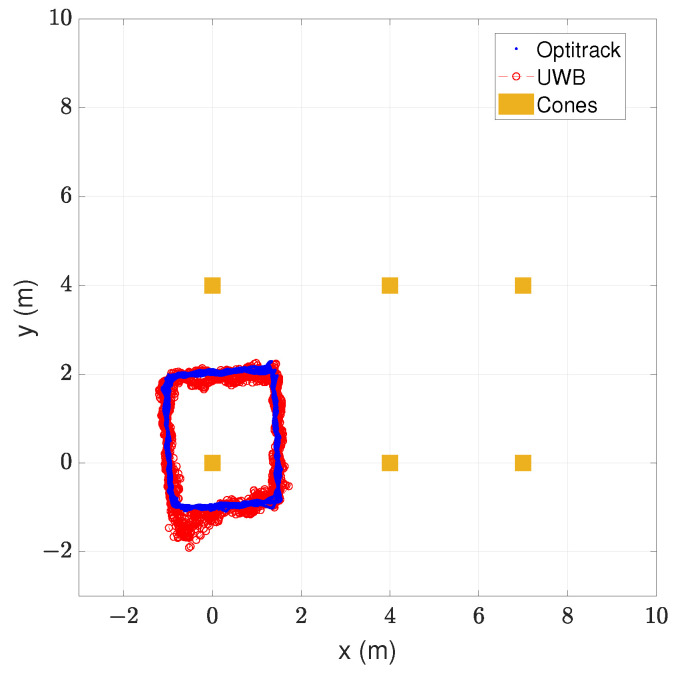
Real vs. estimated trajectory in indoor environment with real cone infrastructure.

In Figure 9, the walked trajectory of the indoor environment can be seen.

The walked path was a rectangle around the cone In-A0 placed on the origin of coordinates. This path was walked six times, three times clockwise and three times counter-clockwise. The blue points represent the measured positions by the ground truth and the red circles the estimated positions by the proposed safety system. In the same picture, the cones’ positions are represented with orange squares. The corresponding metrics of the positioning error are presented in Table 6.

The columns of the table, from left to right, represent the mean positioning error μ, the standard deviation of the positioning error σ, the positioning RMSE, and the maximum positioning error $\epsilon_{max}$. The units of all statistics are metres.

Results in Table 6 show that the position estimates are similar to those of the ground truth. The measured mean positioning error was 0.172 m with a standard deviation of 0.128 m and an RMSE of 0.214 m. The obtained maximum positioning error was 0.983 m, which happened around the bottom-left corner of the walked rectangle. At this place, the estimated position was further away from the anchors’ rectangle than the real position, so an alarm was given anyway.

The positioning accuracy results suggest that the proposed safety system can accurately classify the position of a worker in the previously defined zones. The obtained RMSE was 0.214 m, notably below the average shoulder width of an adult human or the width of the base of a safety cone. Moreover, these results were obtained in spite of the obstacles in the tracking area, such as the tripods and the column, shown in Figure 7b, as well as the NLOS conditions between the tag and anchors in the cones. Note that in these measurements, there was no direct line of sight between anchor In-A4 and the tag because of the column shown in Figure 7b. Due to the realism of the tested environment, the proposed safety system is expected to classify correctly if a worker is in the danger, warning, or safe zone in a real construction environment.

### 5.2. Classification Performance in Indoor Environment Using Real Cone Positions

In order to test the classification performance of our proposed safety system, a person wearing a tag stood at three different points close to one of the limits of the cones’ rectangle, as shown in Table 3. There were 537 measured samples in the danger zone, 544 samples in the warning zone, and 504 samples in the safe zone. The resulting confusion matrix is shown in Table 7.

The safety system classified the tag to be in the correct zone with an accuracy of 96.6% and an $F_{1}$ score of 94.9%. The only misclassified samples were 81 samples in the warning zone detected as in danger. We consider this case safer than misclassifying the tag in the warning zone as safe, since the user of the safety system should not spend too much time in the warning zone. If a worker receives an alarm in the warning zone, they will be aware of the risk of this zone. Moreover, the warning zone had no false positives.

Note that all samples in the safe zone were classified as safe. The absence of false alarms in the safe zone is important not to annoy the worker wearing the tag and maintain their safety commitment. The other way around was also true, as all samples in the danger zone were classified as in danger. This means that an alarm was always given while the person wearing the tag remained in the danger zone, improving their safety awareness.

As a conclusion of the first two experiments in the indoor environment, the proposed safety system proved to be useful to tell with high reliability if a worker is in a safe working zone or not.

### 5.3. Indoor Performance Using Self-Positioned Cones

The proposed safety system has a simple deployment thanks to the self-positioning feature. To evaluate this feature, the calibration tag was placed on each safety cone and the distances to other cones were measured, as described in the procedure of Section 3. At the position of each cone, 20 estimates of the distance to the other cones were calculated. With a ranging rate of 5 Hz, the self-positioning demanded only 4 s per cone. The resulting cone position estimates are shown in Table 8 for the indoor environment.

As can be observed in the table, the cones were self-positioned with two different configurations. The first configuration, In-conf1, fixed the cone In-A0 as the origin of coordinates. The second configuration, In-conf2, fixed the cone In-A3 as the origin of coordinates. Two configurations are presented in this work to show the influence of the chosen origin of coordinates on the performance of the safety system. In the indoor environment, positions were always recorded close to the cone In-A0. If we fix this cone as the origin of coordinates, the positioning accuracy will not be highly influenced by the errors of the estimated cone infrastructure. However, if we fix the cone In-A3, which is the opposite of In-A0, the errors of the estimated cone infrastructure could have a higher influence.

**Figure 10:**
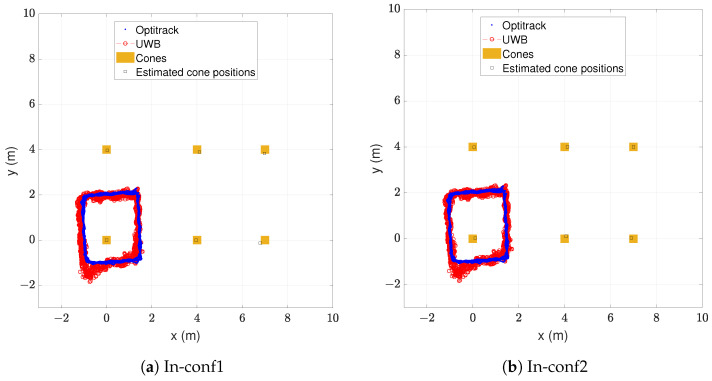
Real vs. estimated trajectory in indoor environment with estimated cone infrastructure.

With the estimated cone positions, a new analysis as in the previous two subsections was made to test the performance of the proposed safety system in the indoor environment. In Figure 10a, the walked real and estimated trajectories can be seen with the cone infrastructure resulting from the configuration In-conf1.

Figure 10b shows the real and estimated trajectories with the cone infrastructure resulting from the configuration In-conf2. The blue points represent the measured positions by the ground truth and the red circles the estimated positions by the proposed safety system. The real cone positions are represented with orange squares and the estimated cone positions with black squares. The corresponding statistics of the positioning error are presented in Table 9.

The obtained positioning accuracy results did not highly differ from the original measurements with the real cone infrastructure. The configuration In-conf1 obtained a mean error of 0.212 m and In-conf2 obtained a mean error of 0.157 m, while the original measurement obtained a mean error of 0.172 m. The reason for these small differences between In-conf1 and In-conf2 lies in the errors that the estimated positions of cones had with different self-positioning configurations, which can be observed in the last column of Table 8. These errors influenced the estimated positions of the tag. In spite of these small differences, we can conclude that the positioning accuracy with the self-positioned cones was still good enough for the proposed application. Moreover, the RMSE value remained, in both configurations, notably below the average shoulder width of an adult human.

In order to confirm the adequacy of the proposed safety system with self-positioned cones, the classification performance was also evaluated with the same tests as in Section 5.2. Table 10 shows the obtained confusion matrices with both configurations of the self-positioning approach of cones.

Both approaches obtained a good classification performance and were almost identical, with an accuracy of 97.0% and an $F_{1}$ score of 95.5%. The few misclassified samples consisted of 72 samples in the warning zone misclassified as in danger in the case of In-conf1, and 71 samples in the warning zone misclassified as in danger in the case of In-conf2.

As discussed in Section 5.2, it is safer to misclassify a worker in the warning zone as in the danger zone than in the safe zone. Moreover, it is important to remark that no samples in the safe zone were misclassified as in danger and no samples in the danger zone were misclassified as safe. Moreover, both self-positioning approaches obtained a slightly better classification performance than the results with the real cone infrastructure. As a consequence, it can be concluded that using the self-positioning approach of cones at least maintains the reliability of the safety system. Not having an accurate piece of equipment to measure the cones’ positions should not be a problem.

### 5.4. Classification Performance in Movement

**Figure 11:**
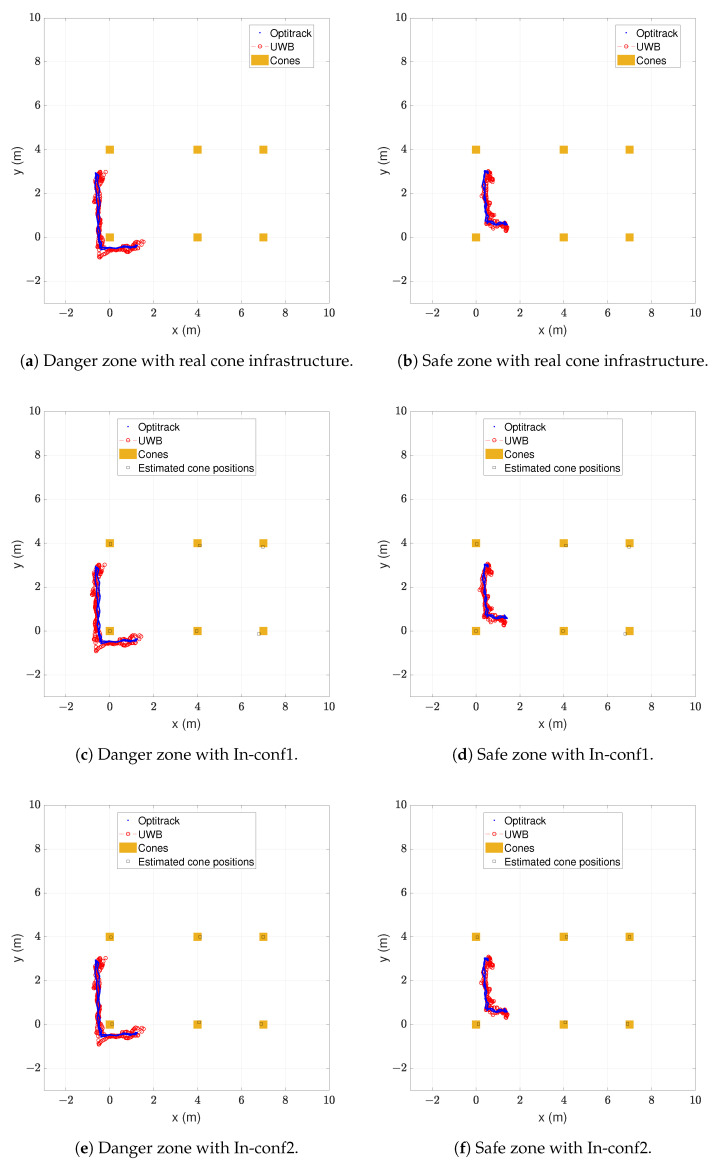
Trajectories walking 0.5 m away from the cones’ limits.

A key performance indicator of our proposed safety system is the number of samples in the danger zone misclassified as safe and samples in the safe zone misclassified as in danger. These misclassified samples were zero in the previous experiments, but only static measurements were taken. Taking advantage of the availability of the Optitrack system in the indoor environment, a 1.78 m tall person wearing a hard hat with a tag walked two different paths, one remaining in the safe zone and the other remaining in the danger zone. In both cases, the tag remained approximately 0.5 m away from the limits of the cones’ rectangle and faced the forward walking direction. With the same two experiments, positions with the real cone infrastructure and the two configurations of the self-positioned cones were obtained. The results are shown in Figure 11.

The blue points represent the measured positions by the ground truth and the red circles the estimated positions by the proposed safety system. The real cone positions are represented with orange squares and the estimated cone positions with black squares. In Table 11, the number of correctly estimated samples is shown for each case of Figure 11.

The majority of samples were estimated to be in their correct zones. With the real cone infrastructure, shown in Figure 11a,b, only a single sample out of 246 taken in the danger zone was misclassified as in the warning zone. All 159 samples of the safe zone were classified as safe. With the estimated cone infrastructure with the configuration In-conf1, shown in Figure 11c,d, all 246 samples in the danger zone were classified as in danger. Only a single sample out of the 159 samples in the safe zone was misclassified as in the warning zone. The classification performance obtained with the estimated cone infrastructure with configuration In-conf2, shown in Figure 11e,f, was identical to that obtained with In-conf1.

As with the previous results in classification performance, no false alarms were given while the tag was in the safe zone and at least one warning was given while the tag was in the danger zone. Having a moving tag did not affect the classification performance. The classification performance was also unchanged with the real and estimated cone infrastructures.

### 5.5. Outdoor Performance

As the proposed safety system had a good performance in the indoor environment, its repeatability was tested by taking some more tests in an outdoor environment. As in the indoor environment, all tests were performed with the real cone infrastructure and two configurations of the self-positioning approach: Out-conf1 and Out-conf2. The estimated cone positions in the outdoor environment can be seen in Table 12.

The configuration Out-conf1 fixed the cone Out-A0 to the origin of coordinates, and the configuration Out-conf2 fixed the cone Out-A2 to its real position, as it was the furthest cone from the measurement zone.

**Figure 12:**
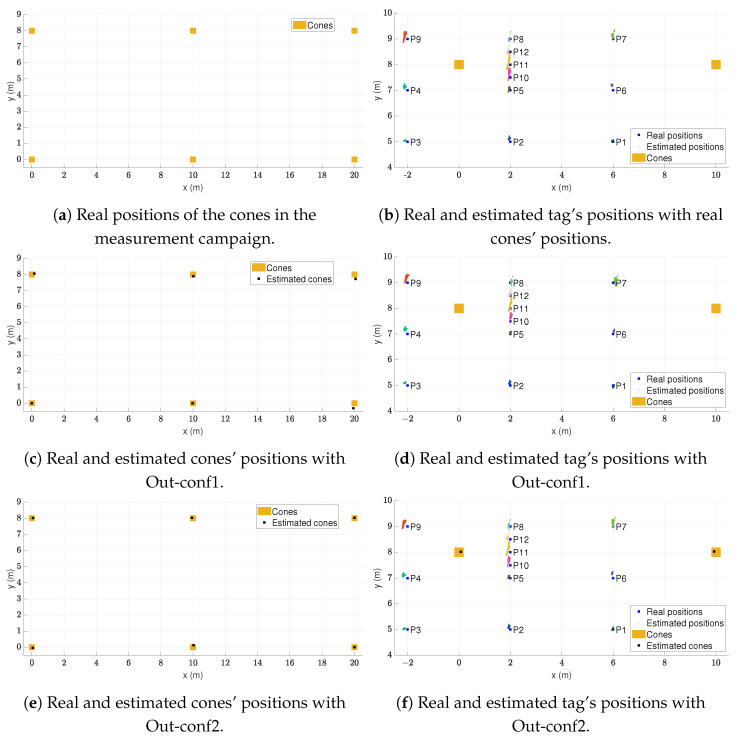
Real vs. estimated measured positions of the proposed safety system outdoors.

As in the indoor environment, first the positioning accuracy was tested. A tag on a tripod was placed on 12 different points inside and outside the cones’ rectangle. Figure 12a,c,e show the area covered by the cones in this measurement campaign and the real and estimated positions of these cones. Figure 12b,d,f present the real and estimated positions of the tag.

The blue squares represent the real positions of the tag and the dots the estimated positions. A different colour is used for the estimated positions at each point. The orange squares represent the positions of the cones. In the cases of estimated cone infrastructure, black squares are added to indicate the estimated cone positions. The corresponding statistics of the measured positioning errors are shown in Table 13, Table 14 and Table 15.

From left to right, the columns of the table contain the following information: name of the measured point, mean positioning error μ, standard deviation of the positioning error σ, positioning RMSE, and maximum positioning error $\epsilon_{max}$. The units of all statistics are metres.

With the ideal case of real cone infrastructure, the positioning accuracy was good enough for the application of the safety system. The mean positioning error was 0.134 m, the standard deviation was 0.069 m, the RMSE was 0.151 m, and the maximum error was 0.466 m. Moreover, all points presented an RMSE notably below the average shoulder width of an adult human.

Comparing the results in Table 13, Table 14 and Table 15, we can observe that the positioning accuracy was not significantly influenced by using the estimated cone positions instead of the real ones. As in the indoor experiments, a satisfactory positioning accuracy was obtained both measuring the real positions of cones or using the self-positioning approach.

In order to evaluate the classification performance, the points Out-P10, Out-P11, and Out-P12 were chosen, as they were the closest ones to a limit. The resulting confusion matrix is shown in Table 16.

Overall, there were 913 measured samples in the danger zone, 845 samples in the warning zone, and 943 samples in the safe zone.

Using the real cone positions, the classification accuracy was 97.5% with an $F_{1}$ score of 96.3%. No samples in the danger zone were misclassified as safe or the other way around. Using the estimated cone positions, both approaches obtained identical results. The classification accuracy was 98.9% with an $F_{1}$ score of 98.3%. Thus, we can conclude that the classification performance of our proposed safety system is similar in both outdoor and indoor environments. Moreover, our proposed safety system proved to have high reliability without the need to measure the positions of cones with extra equipment.

### 5.6. Summary of Results

In Table 17, a summary of the obtained results is shown.

The proposed safety system has shown to have good accuracy with both static and dynamic measurements, so the EKF algorithm works similarly in both types of measurements. The mean positioning error and the RMSE values have always been far below the average shoulder width of an adult human. This positioning accuracy was translated to a highly reliable classification performance, which showed accuracies of at least 96.6%. If the tag was placed in the safe zone, false alarms were never registered. If the tag was in the danger zone, it was never considered to be safe. These results were obtained in two different environments with different deployments, so the results are repeatable. The self-positioning approach was also tested with the safety system, which resulted in a small impact on the classification performance. The proposed safety system using the self-positioning of cones can be as reliable as with known cone positions.

### 5.7. Comparison with State of the Art

Our proposed safety system is compared with the state of art in Table 18.

The reported data in [14,16,17,18,19,20,21,23,24,25,28] have been used in the comparison table, as we have not been able to make this comparison by simulation or real testing. The first two columns of Table 18 contain the used technology in each work and the testing condition (static or dynamic). From the third to the sixth columns, some performance metrics are shown: the presented mean positioning error, the presented positioning RMSE, the presented classification accuracy, and the presented $F_{1}$ score. Finally, the last four columns describe the simplicity of the deployment of the proposed systems. Column 7 indicates whether or not anchors are connected by cables, Column 8 indicates whether or not extra infrastructure is needed, Column 9 indicates whether or not an auto-positioning solution of fixed sensors is proposed, and Column 10 indicates the time to deploy the safety system. Note that not all these data are available in all the works. If a piece of information is not available, this is indicated with N.A. in the corresponding cell.

Compared to the works using BLE and BLE 5.1 [14,16], our proposed safety system obtained better accuracy and $F_{1}$ score. Moreover, our system is simpler to deploy. The solution of [14] needs to put the BLE fixed sensors on the ceiling of a building under construction, while [16] needs to use poles with a height of 10 m. Apart from that, after the deployment of the safety systems, the solutions of [14,16] do not mention any method to simplify the position measurement of fixed sensors.

Although the accuracy of BLE can be improved with the help of inertial sensors [18], our proposed safety system obtained better positioning accuracy. Moreover, the work of [18] is more complex to deploy than our proposal and adds extra elements to the construction site.

Compared to [17], where CSS was used, our proposed safety system obtains lower positioning mean errors thanks to UWB technology. The authors of [17] gave importance to the simplification of the deployment of their system. That is the reason why they avoided using any cabling system among fixed sensors. They also indicate that in the run experiment, two people needed about 13 min to set up the whole system on a construction site. Due to the differences between our tested site and theirs, it is difficult to compare the deployment time of our safety system to theirs. However, an important part of the deployment of [17] consists of moving from one point to the other of the construction site and installing the necessary fixed sensors. In the experiments presented in this work, one minute was needed to perform the deployment of the system. It should be noted that in our case, the time spent placing cones on their positions should not be counted, as safety cones would have been placed anyway. We only need to place the calibration tag, taking 4 s, on each cone to estimate the cones’ positions, which considerably simplifies the deployment.

Compared to most of the works using UWB [19,20,21,23,24,25], our proposed safety system does not need any cabling system among anchors. These cabling systems suppose extra deployment time and increased hazard because of the risk for a worker to trip and fall with one of the cables. The work of [28] successfully removed the cabling system at the expense of reduced accuracy. Compared to [19,20,21,23,24,25], however, our proposed safety system achieves similar accuracy and does not need any cables among anchors.

## 6. Conclusions

In this work, a safety system based on UWB RTLS has been proposed. Our proposal is especially interesting for maintenance works of roads and railways, but it could also be used for any construction site where a safe working environment has to be delimited with cones.

Unlike other proposals, our safety system is deployed easily and adapts quickly to any change in the construction project. Moreover, our hardware is placed inside safety cones, a piece of equipment which is already common on construction sites. Thus, our proposal adds no extra congestion to construction sites and it is unobtrusive. Moreover, the high accuracy of UWB technology makes the system capable of accurately detecting if a worker is in a predetermined safe zone or not.

A typical bottleneck of systems similar to ours is measuring the positions of cones that contain UWB anchors. If the needed equipment is not available to measure the positions of cones, the deployment can be burdensome and error-prone. As a solution, an approach to quickly calculate the positions of cones has been proposed using UWB measurements of available sensors. The proposed self-positioning approach does not require that anchors communicate with each other or that they be connected to a central computer. In this way, the power consumption is reduced and there is no need to add a communication link, which adds preparation time. Moreover, this novel self-positioning approach also calculates automatically the coordinates of the limits of the safe, warning, and danger zones.

The obtained results show that our proposed safety system does not have significant differences in its performance if real or estimated cone positions are used. In any case, the RMSE was always considerably below the average shoulder width of an adult human. Moreover, no false alarms were registered when the tag was in the safe zone, and in the danger zone, a warning or an alarm was always given.

## Figures and Tables

**Table 1:** Configuration parameters of the ultra-wideband (UWB) sensors.

Parameter	Value	Units
Carrier frequency	6.4896	GHz
Bandwidth	499.2	MHz
Channel	5	-
Bitrate	6.8	Mbps
Pulse repetition frequency (PRF)	16	MHz
Preamble length	128	symbols
Preamble code	3	-
Start of frame delimiter (SFD)	8	symbols
Ranging rate	5	Hz

**Table 2:** Real positions of cones in the indoor environment.

Cone	X (m)	Y (m)
In-A0	0.0	0.0
In-A1	4.0	0.0
In-A2	7.0	0.0
In-A3	7.0	4.0
In-A4	4.0	4.0
In-A5	0.0	4.0

**Table 3:** Positions of tested points in the indoor environment for classification performance evaluation.

Point	X (m)	Y (m)	Zone
In-P1	1.5	0.5	Safe
In-P2	1.5	0.0	Warning
In-P3	1.5	−0.5	Danger

**Table 4:** Real positions of cones in the outdoor environment.

Cone	X (m)	Y (m)
Out-A0	0.0	0.0
Out-A1	10.0	0.0
Out-A2	20.0	0.0
Out-A3	20.0	8.0
Out-A4	10.0	8.0
Out-A5	0.0	8.0

**Table 5:** Positions of tested points in the outdoor environment.

Point	X (m)	Y (m)	Zone
Out-P1	6.0	5.0	Safe
Out-P2	2.0	5.0	Safe
Out-P3	−2.0	5.0	Danger
Out-P4	−2.0	7.0	Danger
Out-P5	2.0	7.0	Safe
Out-P6	6.0	7.0	Safe
Out-P7	6.0	9.0	Danger
Out-P8	2.0	9.0	Danger
Out-P9	−2.0	9.0	Danger
Out-P10	2.0	7.5	Safe
Out-P11	2.0	8.0	Warning
Out-P12	2.0	8.5	Danger

**Table 6:** Positioning accuracy of the safety system indoors with real cone infrastructure.

μ (m)	σ (m)	RMSE (m)	$\epsilon_{\max}$ (m)
0.172	0.128	0.214	0.983

**Table 7:** Confusion matrix of the safety system indoors.

		Estimated State		
		Danger	Warning	Safe
Actual state	Danger	537	0	0
	Warning	81	463	0
	Safe	0	0	504

**Table 8:** Real vs. estimated cone positions in indoor environment.

Conf	Cone	Estimated Position		2D Error (m)
		X (m)	Y (m)	
In-conf1	In-A0	0.000	0.000	0.000
	In-A1	3.963	0.000	0.037
	In-A2	6.797	−0.129	0.241
	In-A3	6.975	3.834	0.168
	In-A4	4.101	3.894	0.146
	In-A5	0.033	3.966	0.047
In-conf2	In-A0	0.106	0.022	0.108
	In-A1	4.068	0.104	0.124
	In-A2	6.904	0.034	0.102
	In-A3	7.000	4.000	0.000
	In-A4	4.125	4.000	0.125
	In-A5	0.057	3.988	0.058

**Table 9:** Positioning accuracy of the safety system indoors with self-positioned cones.

Cone Positions	μ (m)	σ (m)	RMSE (m)	$\epsilon_{\max}$ (m)
In-conf1	0.212	0.136	0.252	1.052
In-conf2	0.157	0.126	0.201	0.959

**Table 10:** Confusion matrix of the safety system indoors with self-positioned cones.

Cone Positions	Actual State	Estimated State		
		Danger	Warning	Safe
In-conf1	Danger	537	0	0
	Warning	72	472	0
	Safe	0	0	504
In-conf2	Danger	537	0	0
	Warning	71	473	0
	Safe	0	0	504

**Table 11:** Classification performance in movement.

Zone	Cone Infrastructure	Samples in Correct Zone	Total Samples
Danger	Real	245	246
Safe	Real	159	159
Danger	In-conf1	246	246
Safe	In-conf1	158	159
Danger	In-conf2	246	246
Safe	In-conf2	158	159

**Table 12:** Real vs. estimated cone positions in outdoor environment.

Conf	Cone	Estimated Position		2D Error (m)
		X (m)	Y (m)	
Out-conf1	Out-A0	0.000	0.000	0.000
	Out-A1	9.966	0.000	0.034
	Out-A2	19.940	−0.306	0.312
	Out-A3	20.079	7.716	0.295
	Out-A4	10.007	7.889	0.111
	Out-A5	0.150	8.055	0.160
Out-conf2	Out-A0	0.058	−0.039	0.070
	Out-A1	10.022	0.134	0.136
	Out-A2	20.000	0.000	0.000
	Out-A3	20.000	8.023	0.023
	Out-A4	9.927	8.022	0.076
	Out-A5	0.069	8.018	0.071

**Table 13:** Positioning accuracy of the safety system outdoors with real cone positions.

Point	μ (m)	σ (m)	RMSE (m)	$\epsilon_{\max}$ (m)
Out-P1	0.043	0.009	0.044	0.079
Out-P2	0.113	0.013	0.114	0.225
Out-P3	0.116	0.012	0.116	0.151
Out-P4	0.196	0.023	0.197	0.277
Out-P5	0.095	0.019	0.097	0.147
Out-P6	0.203	0.015	0.204	0.254
Out-P7	0.081	0.041	0.091	0.386
Out-P8	0.054	0.017	0.057	0.300
Out-P9	0.224	0.038	0.227	0.305
Out-P10	0.237	0.053	0.242	0.388
Out-P11	0.136	0.047	0.144	0.466
Out-P12	0.107	0.012	0.108	0.248
All	0.134	0.069	0.151	0.466

**Table 14:** Positioning accuracy of the safety system outdoors with configuration Out-conf1.

Point	μ (m)	σ (m)	RMSE (m)	$\epsilon_{\max}$ (m)
Out-P1	0.057	0.016	0.059	0.105
Out-P2	0.090	0.016	0.092	0.211
Out-P3	0.150	0.009	0.150	0.178
Out-P4	0.218	0.018	0.219	0.307
Out-P5	0.062	0.017	0.065	0.111
Out-P6	0.114	0.017	0.115	0.204
Out-P7	0.107	0.023	0.110	0.348
Out-P8	0.065	0.023	0.069	0.286
Out-P9	0.243	0.039	0.247	0.323
Out-P10	0.207	0.046	0.212	0.343
Out-P11	0.068	0.061	0.091	0.441
Out-P12	0.054	0.019	0.057	0.246
All	0.121	0.071	0.140	0.441

**Table 15:** Positioning accuracy of the safety system outdoors with configuration Out-conf2.

Point	μ (m)	σ (m)	RMSE (m)	$\epsilon_{\max}$ (m)
Out-P1	0.032	0.009	0.033	0.098
Out-P2	0.102	0.013	0.103	0.212
Out-P3	0.143	0.014	0.144	0.183
Out-P4	0.204	0.020	0.205	0.269
Out-P5	0.085	0.011	0.086	0.118
Out-P6	0.182	0.016	0.182	0.261
Out-P7	0.059	0.055	0.081	0.372
Out-P8	0.075	0.017	0.077	0.263
Out-P9	0.220	0.022	0.221	0.281
Out-P10	0.213	0.041	0.217	0.344
Out-P11	0.136	0.042	0.142	0.436
Out-P12	0.121	0.011	0.122	0.251
All	0.131	0.066	0.147	0.436

**Table 16:** Confusion matrix of the safety system outdoors.

Cone Positions	Actual State	Estimated State		
		Danger	Warning	Safe
Real	Danger	913	0	0
	Warning	62	782	1
	Safe	0	38	905
Out-conf1	Danger	913	0	0
	Warning	46	799	0
	Safe	0	0	943
Out-conf2	Danger	913	0	0
	Warning	46	799	0
	Safe	0	0	943

**Table 17:** Summary of obtained results.

Scenario	Cone Infrastructure	μ (m)	σ (m)	RMSE (m)	Accuracy (%)	$F_{1}$ Score (%)
Indoors	Real	0.172	0.128	0.214	96.6	94.9
Indoors	In-conf1	0.212	0.136	0.252	97.0	95.5
Indoors	In-conf2	0.157	0.126	0.201	97.0	95.5
Outdoors	Real	0.134	0.069	0.151	97.5	96.3
Outdoors	Out-conf1	0.121	0.071	0.140	98.9	98.3
Outdoors	Out-conf2	0.131	0.066	0.147	98.9	98.3

**Table 18:** Comparison with state-of-the-art safety systems.

Work	Test	μ (m)	RMSE (m)	Acc (%)	$F_{1}$ (%)	Wired Anchors	Extra Infr.	Auto-Pos.	Time to Deploy (min)
BLE [14]	Dynamic	N.A. ^1^	N.A.	82	88	No	Yes	No	N.A.
BLE 5.1 [16]	Dynamic	N.A.	N.A.	92	84	Yes	Yes	N.A.	N.A.
BLE + IMU [18]	Dynamic	0.320	N.A.	N.A.	N.A.	No	Yes	N.A.	N.A.
CSS [17]	Static	0.868	N.A.	N.A.	N.A.	No	Yes	N.A.	13
UWB [19]	Static	0.087	N.A.	N.A.	N.A.	Yes	Yes	No	N.A.
UWB [20]	Static	N.A.	0.160	N.A.	N.A.	Yes	Yes	N.A.	N.A.
UWB [21]	Dynamic	N.A.	0.229 ^2^	N.A.	N.A.	Yes	Yes	N.A.	N.A.
UWB [23]	Dynamic	0.908	N.A.	N.A.	N.A.	Yes	Yes	No	N.A.
UWB [24]	Static	0.15	N.A.	N.A.	N.A.	Yes	Yes	N.A.	N.A.
UWB [25]	Dynamic	0.3	N.A.	N.A.	N.A.	Yes	Yes	N.A.	N.A.
UWB [28]	Static	0.5	N.A.	N.A.	N.A.	No	Yes	No	N.A.
UWB [this work]	Static	0.129	0.146	98.4	97.6	No	No	Yes	1
UWB [this work]	Dynamic	0.180	0.223	96.9	95.3	No	No	Yes	1
^1^ When a piece of information is missing, it is indicated with N.A. (Not available). ^2^ Mean of RMSE values of different experiments of [21], since the overall RMSE value was not given.									

## Data Availability

The raw data supporting the conclusions of this article will be made available by the authors on request.

## Abbreviations

The following abbreviations are used in this manuscript:

2D	Two-dimensional
AoA	Angle of arrival
BBS	Behaviour-based safety
BLE	Bluetooth low energy
CSS	Chirp spread spectrum
DoF	Degrees of freedom
EKF	Extended Kalman filter
EU-27	European Union of 27 states
IMU	Inertial measurement unit
LOS	Line of sight
MDS	Multidimensional scaling
NLOS	Non-line of sight
PPE	Personal protective equipment
PRF	Pulse repetition frequency
RFID	Radio frequency identification
RMSE	Root mean square error
RTLS	Real-time locating system
SFD	Start of frame delimiter
SLAM	Simultaneous localisation and mapping
TDMA	Time-division multiple access
TDoA	Time difference of arrival
ToA	Time of arrival
TWR	Two way ranging
UWB	Ultra-wideband
